# Effect of a Polyhexanide-Based Antiseptic Composition on Dentin Microhardness and Mechanical Properties: An In Vitro Study

**DOI:** 10.3390/ma18122900

**Published:** 2025-06-19

**Authors:** Zurab Khabadze, Yulia Generalova, Oleg Mordanov

**Affiliations:** Department of Operative Dentistry, Institute of Medicine, Peoples’ Friendship University of Russia Named after Patrice Lumumba (RUDN University), Miklukho-Maklaya Str. 6, 117198 Moscow, Russia; khabadze-zs@rudn.ru (Z.K.); generalova-yua@rudn.ru (Y.G.)

**Keywords:** dentin microhardness, Young’s modulus, polyhexanide, sodium hypochlorite, EDTA, root canal irrigants, elastic deformation energy

## Abstract

The effect of root canal irrigants on the mechanical properties of dentin is crucial in endodontic treatment planning. While antiseptics such as sodium hypochlorite and EDTA are widely used, their potential to weaken dentin structure remains a concern. Polyhexanide-based formulations may offer a safer alternative. To assess the impact of a polyhexanide-based antiseptic composition, compared to standard irrigants, on the microhardness, Young’s modulus, and elastic deformation energy of dentin. Sixty extracted human teeth were sectioned and polished to prepare dentin samples. Baseline measurements of Vickers microhardness, Young’s modulus, and elastic deformation work were performed using a Microhardness Tester (CSM Instruments, Switzerland) with a Berkovich indenter. Samples were then divided into six groups (n = 10 per group) and exposed to different irrigants (NaCl 0.9%, NaOCl 3%, chlorhexidine 2%, EDTA 17%, and polyhexanide-based solutions—0.1% and 0.2% Lavasept). Post-treatment measurements were performed. Statistical analysis was conducted using non-parametric tests with Bonferroni correction. Sodium hypochlorite (3%) caused the most pronounced reduction in dentin microhardness and mechanical strength, though not always statistically significant. Polyhexanide-based solutions (0.1% and 0.2% Lavasept) showed a milder effect, with statistically significant changes observed only in elastic deformation energy for 0.2% polyhexanide. EDTA treatment led to severe surface destruction, precluding reliable post-treatment measurements. Polyhexanide-based irrigants demonstrated a more favorable impact on dentin mechanical properties compared to traditional irrigants, supporting their potential use in endodontic protocols aimed at preserving dentin integrity.

## 1. Introduction

The mechanical properties of root dentin play a critical role in ensuring the long-term success of endodontically treated teeth. Various chemical irrigants used during root canal procedures, such as sodium hypochlorite (NaOCl) and ethylenediaminetetraacetic acid (EDTA), have been shown to adversely affect dentin by altering its hardness, elasticity, and microstructural integrity [1,2]. These changes can weaken the tooth structure and increase the risk of root fractures after treatment [3].

In response to these concerns, alternative irrigants with improved biocompatibility have been explored. Polyhexamethylene biguanide (polyhexanide, PHMB) has emerged as a potential candidate due to its broad-spectrum antimicrobial activity and reduced cytotoxic potential. However, despite its promising biological profile, there is no information regarding the influence of polyhexanide-based solutions on the mechanical properties of dentin, including microhardness and elasticity. Previous research into natural irrigants, such as propolis, and newer formulations like Chloroquick (a combination of NaOCl and etidronic acid), highlight the need for gentler agents that minimize adverse effects on dentin [4,5].

Dentin is a key structural component of the tooth, exhibiting unique mechanical properties that ensure its function and resistance to physiological load. One of the essential characteristics is dentin microhardness, which normally ranges from 50 to 70 Vickers hardness number (HV), depending on anatomical zone and method of measurement [6]. For example, near the dentin–enamel junction (DEJ), the average hardness is approximately 55.78 ± 2.88 HV [6].

The Young’s modulus (elastic modulus) of dentin also varies considerably: in peritubular dentin, it may reach values of 40–42 GPa, while in intertubular regions it is generally around 20–25 GPa [7]. This difference is primarily due to structural and mineralization gradients across dentin zones [7,8].

Chemical irrigants used during endodontic therapy have been shown to significantly alter the mechanical properties of dentin. A recent systematic review and meta-analysis confirmed that prolonged contact with irrigants, particularly 2.5% sodium hypochlorite (NaOCl), leads to a marked decrease in dentin microhardness [8]. The use of chelating agents such as EDTA and acidic solutions can further reduce both microhardness and the elastic modulus of dentin [9,10]. These changes may adversely affect the long-term structural integrity of the tooth and its resistance to functional loading following root canal therapy.

Also, previous studies have demonstrated that NaOCl can cause deproteinization of the organic matrix within dentin, leading to a significant decrease in its elastic modulus and microhardness [6]. Similarly, although EDTA is effective in removing the smear layer, it may cause excessive demineralization of dentin, resulting in compromised mechanical strength [11,12]. Furthermore, it has been shown that higher concentrations and prolonged exposure to NaOCl worsen the degradation of dentin’s organic matrix [13,14,15].

The aim of this study was to evaluate and compare the effects of polyhexanide-based antiseptic compositions and traditional endodontic irrigants on the mechanical properties of root dentin, specifically microhardness, Young’s modulus, and elastic deformation energy, using a nanoindentation method. Two null hypotheses were proposed: (H_01_) there are no statistically significant differences in dentin microhardness, Young’s modulus, or elastic deformation energy among the groups treated with different irrigants; and (H_02_) polyhexanide-based solutions have a similar impact on dentin mechanical properties compared to conventional irrigants such as sodium hypochlorite and EDTA.

## 2. Materials and Methods

### 2.1. Sample Preparation

The study was conducted in accordance with the Declaration of Helsinki and approved by the Local Ethics Committee of federal state autonomous educational institution of higher education “Peoples’ Friendship University of Russia named after Patrice Lumumba” (protocol No. 23 dated 21 December 2023, Moscow, Russia).

The present study included 60 human teeth extracted for orthodontic reasons (maxillary and mandibular third molars and first premolars). Prior to the experiment, all teeth underwent a primary screening process to exclude samples with root fractures, carious lesions, or previous endodontic treatments. The selected teeth were stored in an isotonic solution containing gentamicin sulfate at +4 °C until further use.

The teeth were decoronated using a diamond fissure bur (FO-11 ISO 299/013, Mani, Tochigi, Japan) and a high-speed handpiece (EXPERTtorque E680 L, Kerr, Germany) mounted on a dental unit (Planmeca Compact i Classic, Helsinki, Finland). The decoronated samples were stored in isotonic saline containing gentamicin sulfate until sectioning.

Transverse dentin sections were prepared at the level of the coronal pulp chamber. Slices of 4 mm thickness were obtained using an abrasive cutting machine (PRESI Mecatome T200A, PRESI, Marnaz, France) equipped with an aluminum oxide disk (0.5 mm thickness, 3200 rpm) under continuous cooling with distilled water, resulting in 60 dentin slices.

For standardization and ease of polishing, two slices were embedded in each round plastic mold (30 mm diameter) using freshly prepared modified two-component epoxy resin (ULTIMA Epoxy Adhesive, Magicrete Building Solutions, Surat, India). The resin blocks were allowed to cure at room temperature (20–22 °C) for 24 h, resulting in 30 specimens.

**Figure 1:**
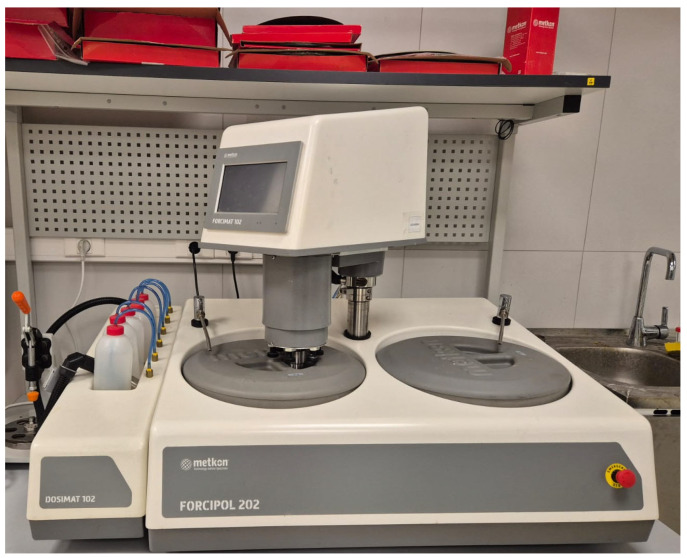
Grinding and polishing system FORCIPOL 202 with FORCIMAT 102 and DOSIMAT 102 (Metkon, Turkey).

After curing, the surfaces were mechanically polished using a grinding–polishing unit (FORCIPOL 202 with FORCIMAT 102 and DOSIMAT 102, Metkon, Bursa, Turkey) to achieve a smooth, flat surface suitable for accurate microhardness testing (Figure 1).

The polishing protocol included:

- **Stage 1 (Backside leveling):** P800 abrasive paper, counter-rotational mode, 6 min, disk speed 200 rpm, head speed 50 rpm, individual load 15 N, water cooling.
- **Stage 2 (Investigated side grinding):** P1200 abrasive paper, 2 min, counter-rotational mode, same parameters as Stage 1.
- **Stage 3 (Polishing):** Silk cloth with 1 µm monocrystalline diamond suspension, 6 min, unidirectional rotation, disk speed 200 rpm, head speed 50 rpm, individual load 10 N, dry polishing.
- **Stage 4 (Final cleaning):** Immersion of samples in chemically pure isopropyl alcohol (Propan-2-ol).

All procedures were carried out at room temperature (22 ± 1 °C). No active humidification was applied during treatment, but the samples were stored in a closed humid chamber (>95% humidity) between irrigation and testing to minimize dehydration.

### 2.2. Microhardness Measurement and Chemical Treatment of Samples

Prior to testing, the operator was trained and calibrated using a standard reference polymer material with known elastic modulus and hardness (ISO standard sample, certified by Manufacturer), achieving intra-operator variability below 5% across three pilot indentations.

Initial microhardness testing was performed using a Microhardness Tester equipped with an automatic optical microscope ConScan 3D (CSM Instruments, Peseux, Switzerland). A Berkovich diamond indenter was used. The indentation depth was kept below 10% of the sample thickness to minimize substrate effects.

Microhardness (H) was calculated according to the Formula (1):

(1) $$H=\frac{P}{A_{C}},$$

where P is the applied load (N) and A_c_ the contact area (m^2^).

The contact depth (h_c_) was determined using (2):

(2) $$h_{c}=h_{max}-\varepsilon C\cdot \frac{Fmax}{S},$$

where S is the contact stiffness, and εC is a geometric constant for the Berkovich indenter (0.75).

The conversion of hardness values measured with the Berkovich indenter (HB) to the Vickers equivalent (HV) was performed using the following Formula (3):

(3) $$HV=\frac{HB}{0.009807}$$

The elastic modulus (Young’s modulus, EIT) was calculated by (4):

(4) $$EIT=\beta \frac{2}{\sqrt{\pi }}E_{r\sqrt{A_{C}}},\;$$

where β is a correction factor (1034 for Berkovich indenters).

The relative elastic work (We) was calculated by (5):

(5) $$We=\frac{\left(h_{m}-h_{n}\right)}{h_{m}}\times 100\%,$$

where h_m_ is the maximum indentation depth and h_n_ is the residual depth after unloading.

Indentation was performed dynamically with a maximum load of 1 N, loading/unloading rates of 2000 mN/min, an approach speed of 16,800.001 nm/min, and a dwell time of 15 s at maximum load. Five consecutive indentations were made on each specimen from the canal outward across the dentin.

After baseline measurements, the samples were randomly assigned to six groups (n = 10 slices per group, 5 blocks per group). The sample size (n = 10 per group) was determined based on power analysis using preliminary data, assuming a 95% confidence level, 80% statistical power, and an expected effect size (Cohen’s d) of at least 1.2 for dentin hardness differences. This yielded a minimum of 8 samples per group; to enhance reliability, 10 specimens were included in each group.

During irrigation, each dentin block was fully immersed in 5 mL of the assigned solution under constant gentle agitation on an orbital shaker (100 rpm) to simulate dynamic clinical exposure. Solutions were freshly prepared and not reused between samples.

Irrigation protocol:

- **Group 1:** 0.9% Sodium chloride solution for 1 h.
- **Group 2:** 3% Sodium hypochlorite (Belodez, VladMiVa, Belgorod, Russia) for 1 h.
- **Group 3:** 2% Chlorhexidine digluconate solution (Omega-Dent, Moscow, Russia) for 1 h.
- **Group 4:** 17% EDTA solution (MD Cleanser, META, Cheongju, Republic of Korea) for 1 h.
- **Group 5:** 0.1% Lavasept (polyhexanide-based solution) for 1 h.
- **Group 6:** 0.2% Lavasept (polyhexanide-based solution) for 1 h.

Polyhexanide solutions were freshly prepared by diluting a 20% stock solution (Lavasept, B. Braun Melsungen AG, Hessen, Germany) with distilled water. The concentrations of Lavasep 0.1% and 0.2% were chosen because they are the manufacturer’s recommended concentrations and are also registered and approved for clinical use in the Russian Federation.

Lavasept contains polyhexamethylene biguanide hydrochloride (polyhexanide) as the active ingredient and the following auxiliary ingredients: macrogol-4000, and water for injection (pH 5.0–7.0).

After treatment, the specimens were rinsed with distilled water, lightly blotted, and air-dried at room temperature (22 ± 1 °C, ~40% humidity) for 15 min in a dust-protected laminar flow cabinet before second seria indentation.

Post-treatment microhardness measurements were performed under the same conditions as the initial assessment. Optical images and load–unload graphs were recorded for analysis.

Data collection and analysis were conducted using Indentation Software Version 4.37 (CSM Instruments, Switzerland) and standard manual calculations.

### 2.3. Statistical Analysis

All quantitative data were analyzed using the software package OriginPro 2023 (OriginLab Corporation, Northampton, MA, USA) and Microsoft Excel 2019 (Microsoft Corporation, Redmond, WA, USA). The normality of data distribution was assessed using the Shapiro–Wilk test. Given the non-normal distribution of the majority of datasets, non-parametric statistical methods were applied. Comparisons between groups were performed using the Mann–Whitney U test. For multiple comparisons, Bonferroni correction was applied to adjust the level of statistical significance. A *p*-value of < 0.05 was considered statistically significant after correction. Quantitative data are presented as mean ± standard deviation (SD). Graphical representations were constructed based on aggregate group data for initial and post-treatment measurements of Vickers microhardness, Young’s modulus, and relative elastic deformation work.

## 3. Results

The physical and mechanical properties of dentin, particularly microhardness, play a crucial role in predicting a tooth’s resistance to mechanical loads. In addition to their effects on microorganisms and the smear layer, irrigating solutions can also influence the structural integrity of root canal wall dentin. A decrease in dentin hardness and elasticity after chemical treatment may lead to unfavorable clinical outcomes. Therefore, it is essential to evaluate the impact of root canal irrigants on the mechanical properties of dentin to optimize irrigation protocols.

In this study, several physical parameters were evaluated, including Vickers microhardness, Young’s modulus, and relative elastic deformation work. Indentational hardness reflects the material’s ability to resist irreversible deformation or fracture under load. Microhardness was assessed under a maximum load of 1 N, consistent with standard microindentation protocols. Calculations were based on the Vickers method (HV), relating the applied load to the contact area of the indentation produced by a Berkovich-type diamond tip. The results obtained from the instrumented indentation were converted to Vickers hardness values using an established geometric correlation between the Berkovich and Vickers indenters.

Each specimen was subjected to initial microhardness measurement, followed by treatment with one of the irrigants:

- Group 1: 0.9% sodium chloride,
- Group 2: 3% sodium hypochlorite (NaOCl),
- Group 3: 2% chlorhexidine digluconate,
- Group 4: 17% EDTA,
- Group 5: 0.1% Lavasept (polyhexanide-based solution),
- Group 6: 0.2% Lavasept (polyhexanide-based solution).

**Figure 2:**
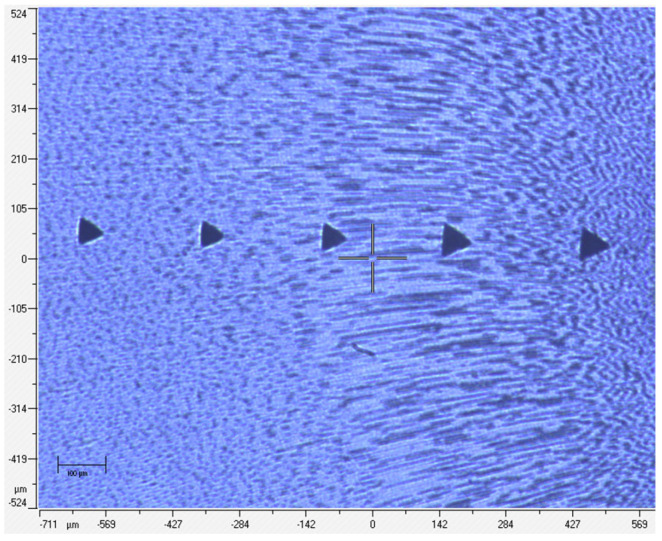
Position of the first series of five indentations before chemical treatment. Specimen number 1 from group 1 (0.9% NaCl).

**Figure 3:**
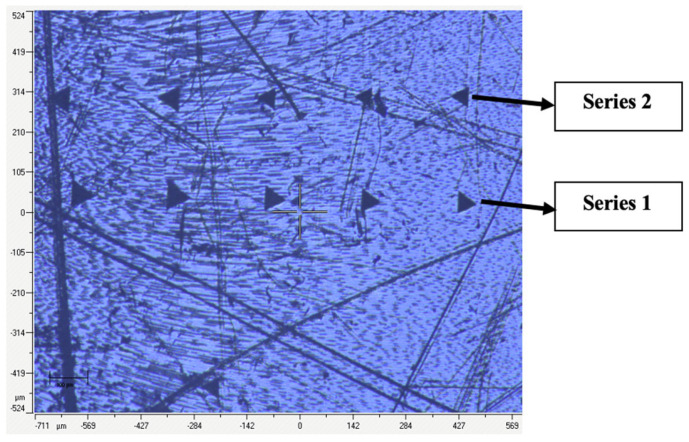
Position of the second series of five indentations after chemical. “Series 1” refers to pre-treatment indentations; “Series 2” refers to post-treatment indentations. Specimen number 1 from group 1 (0.9% NaCl).

**Figure 4:**
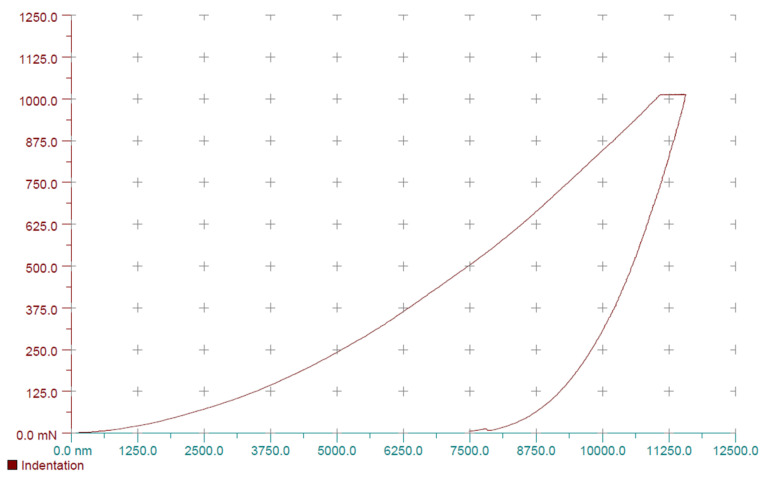
Example of a load–unload curve from the initial microhardness measurement. Specimen number 1 from group 1 (0.9% NaCl).

For each experimental stage (before and after irrigation), 10 indentations per slice were recorded (Figure 2 and Figure 3), along with load–unload curves reflecting deformation and elasticity characteristics (Figure 4).

**Figure 5:**
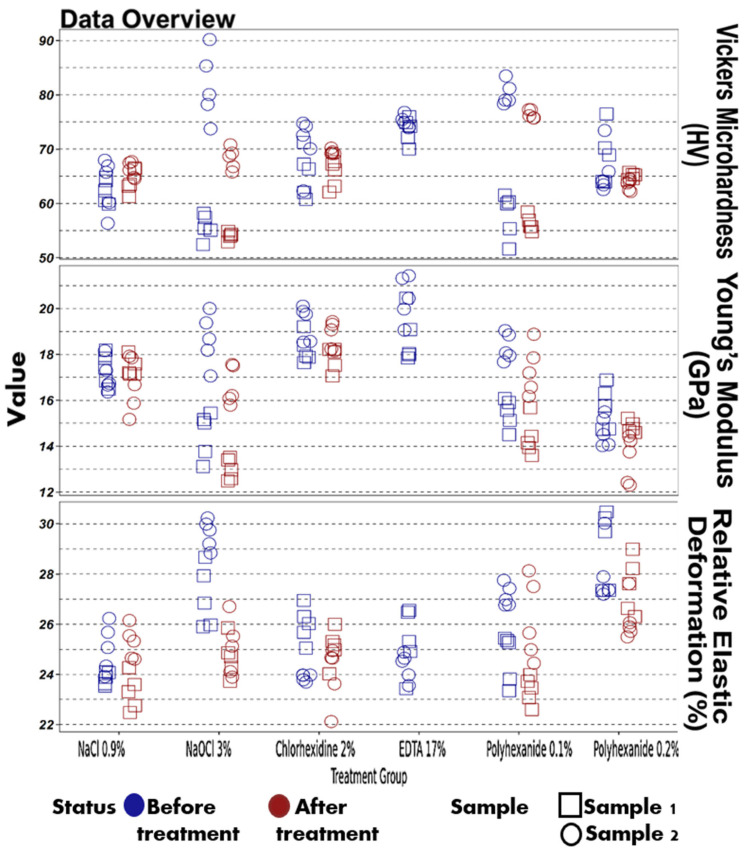
Aggregate data on the effects of the polyhexanide-based antiseptic composition and standard irrigants on the mechanical properties of dentin, based on the first two specimens from each experimental group.

**Figure 6:**
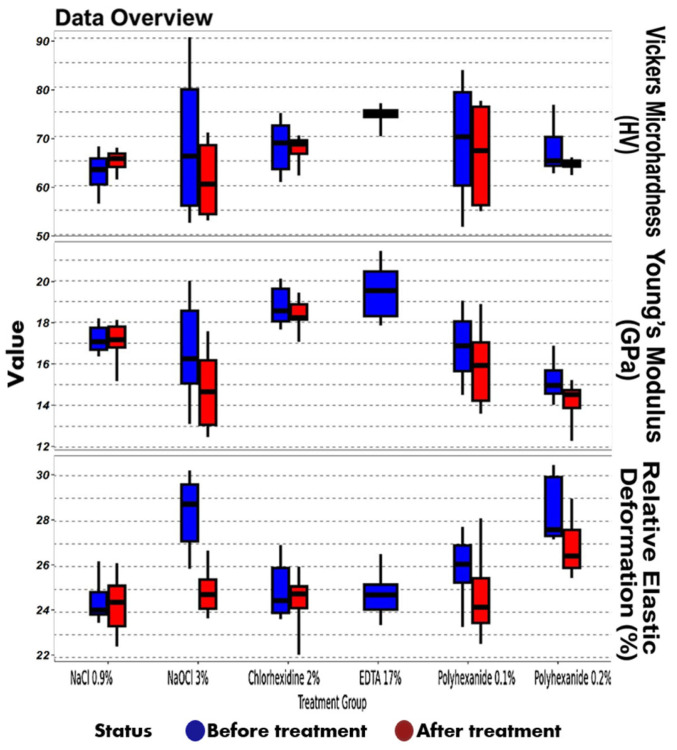
Boxplot distributions of Vickers microhardness (HV), Young’s modulus (GPa), and relative elastic deformation work (%) in dentin samples before and after chemical treatment. Six experimental groups are shown: 0.9% NaCl, 3% NaOCl, 2% chlorhexidine, 17% EDTA, 0.1% polyhexanide, and 0.2% polyhexanide. Blue boxplots represent values before treatment; red boxplots represent values after treatment.

**Figure 7:**
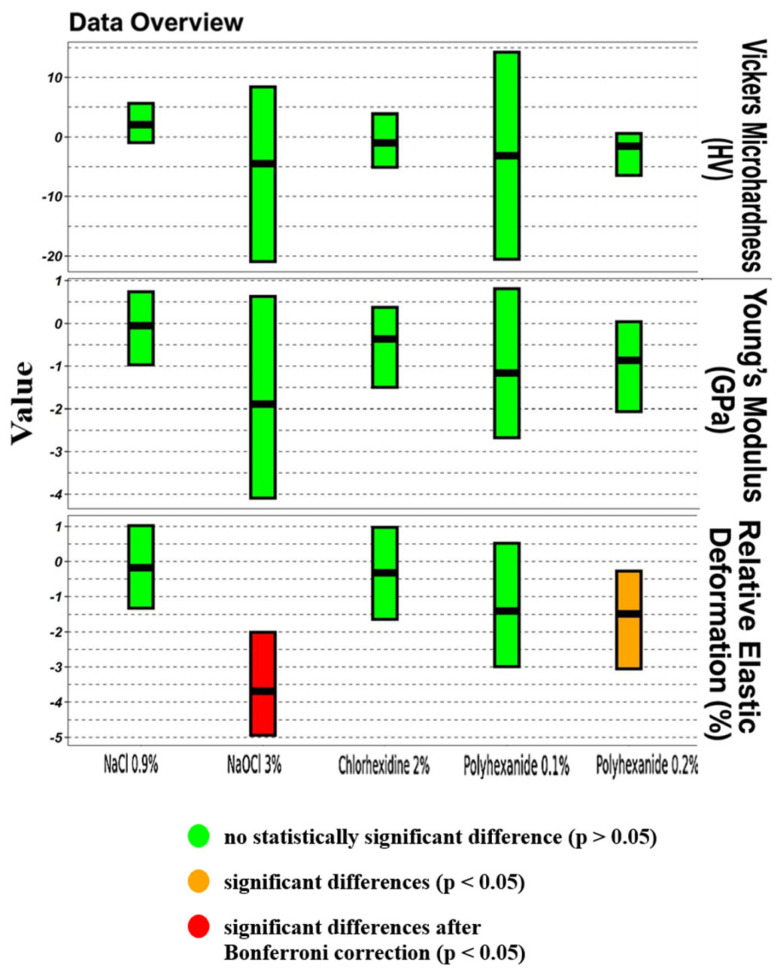
Differences in Vickers microhardness (HV), Young’s modulus (GPa), and relative elastic deformation work (%) in dentin samples after chemical treatment compared to baseline. Green bars indicate no statistically significant difference (*p* > 0.05); red bars indicate significant differences (*p* < 0.05); orange bars indicate significant differences after Bonferroni correction (*p* < 0.05).

Aggregate data regarding the effects of polyhexanide-based and conventional irrigants on dentin microhardness and associated mechanical properties are presented in Table 1, with corresponding graphical representations. Comparative analysis of mechanical properties before and after treatment is shown in Table 2 and visualized in Figure 5, Figure 6 and Figure 7.

Regarding Vickers microhardness, specimens exposed to 0.9% NaCl exhibited a slight tendency toward increased hardness after treatment; however, the differences were not statistically significant (*p* > 0.05). In contrast, all other irrigants resulted in a decrease in microhardness, most pronounced in the 3% NaOCl group, although again without reaching statistical significance. Nevertheless, the reduction observed with NaOCl was greater compared to other groups.

Evaluation of changes in Young’s modulus revealed a similar trend toward decreased elasticity across most experimental groups following chemical treatment. The most significant reduction was again noted in the 3% NaOCl group; however, the differences did not achieve statistical significance (*p* > 0.05), suggesting a potential but unconfirmed negative impact of sodium hypochlorite on dentin elasticity.

The analysis of relative elastic deformation work demonstrated the most prominent changes among all measured parameters. All treated groups showed a reduction in this index post treatment. Statistically significant reductions were recorded in the 0.2% Lavasept group, and even more prominently in the 3% NaOCl group, with *p*-values < 0.05 after Bonferroni correction. These findings indicate that sodium hypochlorite exerts a strong adverse effect on dentin’s ability to undergo elastic deformation.

Of particular note, treatment with 17% EDTA resulted in such severe surface degradation that secondary microhardness measurements could not be performed. Due to the formation of deep surface porosities and irregularities, indentations were incomplete and asymmetric, precluding accurate assessment. Although quantitative data for the EDTA group were excluded from statistical analysis, the extent of surface destruction itself suggests a significant reduction in microhardness.

Regarding the polyhexanide-treated groups, both concentrations (0.1% and 0.2% Lavasept) demonstrated only modest effects on dentin microhardness. The 0.2% Lavasept group exhibited slightly greater reductions; however, differences remained statistically insignificant (*p* > 0.05), except for relative elastic deformation work, where statistically significant differences were detected (*p* < 0.05). These results indicate a minor interaction between 0.2% Lavasept and dentin surface, although its impact is notably lower compared to sodium hypochlorite.

## 4. Discussion

The primary objective of this study was to investigate how various root canal irrigants—particularly polyhexanide-based compositions (Lavasept)—affect the mechanical integrity of root dentin. This is highly relevant in clinical endodontics, where maintaining the physical properties of dentin post treatment is essential for long-term tooth viability. A significant challenge remains in finding irrigants that offer effective disinfection without compromising dentin microstructure. Our findings indicate that among the solutions tested, sodium hypochlorite caused the most aggressive reduction in dentin strength, while polyhexanide showed comparatively gentler effects.

The results partially rejected the first null hypothesis (H_01_), particularly for relative elastic deformation work. Statistically significant differences were observed between baseline and post-treatment values for sodium hypochlorite and 0.2% Lavasept. Although reductions in Vickers microhardness and Young’s modulus were evident across groups, they did not reach statistical significance in most cases, suggesting a more subtle mechanical shift. These findings align with the earlier literature reporting non-linear and complex effects of chemical agents on dentin [3,6].

The second null hypothesis (H_02_), which presumed that polyhexanide would behave similarly to traditional irrigants, was also rejected. Notably, 0.2% Lavasept did induce a statistically significant reduction in relative elastic deformation work, albeit less severe than that of sodium hypochlorite. This outcome supports the hypothesis that polyhexanide possesses a more biocompatible profile, but is not entirely inert to dentin’s mechanical parameters. The reduced impact compared to EDTA and NaOCl makes it a promising candidate for further study.

Sodium hypochlorite remains a cornerstone in endodontics for its unparalleled antimicrobial efficacy and tissue-dissolving capabilities. However, its interaction with the collagen matrix of dentin has been shown to lead to deproteinization, thus reducing elasticity and hardness [1,3,6]. In our study, the reduction in Young’s modulus and Vickers hardness, though statistically non-significant, followed this known trend. More importantly, a marked decline in relative elastic work was statistically significant, indicating impaired ability of dentin to recover from deformation.

EDTA, a widely used chelating agent, displayed destructive effects in our experiment, consistent with previous research highlighting its demineralizing capacity [16,17]. Post-treatment measurements in the EDTA group were impossible due to pronounced surface erosion and structural collapse, which rendered the dentin unsuitable for indentation. Although not quantified statistically, this qualitative observation strongly implies extensive mechanical degradation. Such adverse effects challenge the routine use of EDTA in prolonged or repeated applications.

Chlorhexidine digluconate, another common irrigant, exhibited minimal impact on all measured parameters. Its relatively neutral effect supports its current status as a supplementary antimicrobial agent rather than a primary irrigant. No statistically significant differences in any property were found between pre- and post-treatment values, reinforcing the notion that chlorhexidine is mechanically safer for dentin. However, its limited smear layer removal capacity may restrict its standalone use.

The performance of polyhexanide was of particular interest, especially given its recent introduction into dental applications. Both 0.1% and 0.2% Lavasept concentrations preserved Vickers hardness and Young’s modulus better than NaOCl and EDTA. Only the 0.2% concentration caused a statistically significant reduction in elastic deformation work, suggesting a concentration-dependent interaction. These findings are consistent with earlier reports demonstrating the low cytotoxicity and gentle action of polyhexanide on dentin surfaces [18,19].

The physical explanation behind these differences lies in the chemical nature of each irrigant. Sodium hypochlorite induces collagen degradation, while EDTA chelates calcium ions, resulting in matrix weakening. Polyhexanide, in contrast, exhibits minimal affinity for dentin’s organic or inorganic components, allowing it to disinfect without structural disruption. This distinction is essential when designing irrigation protocols that prioritize both disinfection and preservation [20,21,22].

The methodological rigor of the present study—such as standardized nanoindentation protocols and statistical corrections—strengthens the validity of the results. The use of a Berkovich indenter and dynamic loading ensured reproducible and clinically relevant data. Moreover, the application of Bonferroni correction minimized the risk of Type I errors in multiple comparisons [23,24]. Nonetheless, the sample size per group (n=10) could limit the generalizability of findings

An additional strength of this study is its simultaneous assessment of three mechanical parameters: Vickers microhardness, Young’s modulus, and relative elastic deformation work. This multifaceted approach offers a comprehensive view of dentin’s load-bearing capacity and its capacity for recovery under stress. Of these, relative elastic deformation work emerged as the most sensitive indicator of chemical impact. Future studies may benefit from prioritizing this parameter when evaluating biocompatibility [17,25,26].

A key observation from this study is that polyhexanide-based solutions exhibited a significantly milder impact on dentin compared to traditional irrigants. This aligns with recent SEM-based findings that show 0.1% and 0.2% Lavasept caused less smear layer removal than EDTA or sodium hypochlorite, indicating a gentler interaction with dentin surfaces [27]. These differences may be explained by polyhexanide’s lower affinity for calcium chelation or collagen dissolution, which preserves both inorganic and organic components more effectively than its counterparts.

Sodium hypochlorite, by contrast, is known for its aggressive deproteinizing action. Multiple studies confirm that NaOCl removes essential proteins from dentin’s collagen matrix, contributing to loss of elasticity and hardness even at moderate levels [1,3]. These chemical alterations are believed to degrade the dentin’s structural matrix, potentially increasing the risk of fractures during post-endodontic loading.

EDTA, though crucial for smear layer removal, has consistently been shown to demineralize dentin and significantly reduce microhardness due to its strong calcium-chelating properties [17]. The destructive interaction with inorganic dentin components often results in surface erosion and a collapse of the dentinal tubule structure, a phenomenon corroborated by our inability to perform mechanical tests post-EDTA exposure in this study.

Beyond antimicrobial and physicochemical properties, the impact of irrigants on the mechanical integrity of root dentin is a crucial factor influencing the longevity of endodontic therapy. In our study, exposure to sodium hypochlorite and EDTA resulted in a decrease in dentin microhardness and elastic modulus, which corroborates findings by Perdigão et al. and Pashley et al. [9,10]. These agents, while effective in smear layer removal, may compromise the structural resilience of dentin through demineralization and collagen disruption.

In contrast, polyhexanide-based irrigation demonstrated a more favorable profile, with reduced impact on microhardness, aligning with recent work by Łapińska et al. [8], who emphasized the importance of dentin-preserving disinfection protocols. Previous data suggest that dentin microhardness reductions of more than 25% can impair the fracture resistance of roots [28]. Therefore, irrigants such as polyhexanide, which balance antimicrobial efficacy and mechanical safety, may offer clinical advantages in complex endodontic cases. This highlights the necessity of evaluating both biological and biomechanical outcomes when developing or selecting irrigants for clinical use.

Interestingly, when polyhexanide was evaluated in combination protocols, such as with EDTA, it showed chemical compatibility and did not produce negative interactions like precipitate formation, unlike chlorhexidine or sodium hypochlorite [13]. This suggests potential for using polyhexanide in sequential irrigation regimens that combine effective smear layer removal with mechanical preservation. Future studies could explore this strategy as a viable clinical protocol.

Lastly, the reduction in collagen density noted with polyhexanide exposure was less pronounced than that caused by sodium hypochlorite in recent histological studies [29]. These data reinforce the hypothesis that polyhexanide has a more conservative effect on the dentin matrix, making it an attractive option for patients with structurally compromised teeth where long-term mechanical retention is critical.

Another area for future investigation is the combination of irrigants. For example, alternating polyhexanide with chelating agents in sequential irrigation cycles might provide both smear layer removal and mechanical preservation. Recent formulations, such as etidronate-based solutions or Chloroquick, have attempted this dual approach [5], but polyhexanide has not yet been systematically explored in this context.

It is also worth considering the biological effects of polyhexanide beyond mechanical properties. Previous research suggests that polyhexanide may reduce inflammation and promote favorable healing in periapical tissues [18]. Integrating mechanical and histological analyses could offer a more complete profile of its clinical applicability. Such interdisciplinary approaches are becoming increasingly relevant in modern endodontics.

From a clinical perspective, the evidence presented here supports a more conservative approach to irrigant selection. Polyhexanide, especially in lower concentrations, may allow clinicians to achieve antimicrobial control with reduced risk to dentin integrity. This is particularly important in retreatment cases or structurally compromised teeth where preserving mechanical strength is paramount. However, broader adoption will require additional clinical trials and regulatory approvals.

In summary, this study contributes to a growing body of literature emphasizing the importance of balancing efficacy and biocompatibility in endodontic irrigation. While sodium hypochlorite remains an effective antimicrobial agent, its impact on dentin strength cannot be overlooked. Polyhexanide-based irrigants (Lavasept) offer a promising alternative with milder mechanical effects, although their long-term behavior and interaction with other endodontic materials warrant further research.

This study has several limitations that should be acknowledged. First, the in vitro design may not fully replicate the complex conditions of the oral environment, such as temperature fluctuations, mechanical loading, and continuous microbial interactions. Second, while dentin microhardness and morphological changes were assessed, the evaluation of long-term mechanical fatigue and fracture resistance under dynamic conditions was beyond the scope of this work. Third, the tested irrigants were applied under standardized exposure times, which may not reflect variability in clinical protocols. All irrigants were tested in a single exposure session, which does not simulate the cumulative effects that may occur clinically. Finally, the sample size, although statistically sufficient, limits broader generalizability, and future studies involving clinical samples and patient-centered outcomes are warranted to confirm these results. Another limitation is the exclusion of cyclic or long-term aging models. Incorporating artificial aging protocols would allow a more robust prediction of long-term outcomes.

## 5. Conclusions

This study demonstrated that chemical irrigation agents can influence the mechanical properties of root dentin, with varying degrees of impact depending on the type of solution used. Sodium hypochlorite (NaOCl) caused the most pronounced reductions in microhardness, Young’s modulus, and relative elastic deformation work, while polyhexanide-based solutions showed a more conservative profile. Although the polyhexanide-based antiseptic composition Lavasept at 0.2% concentration caused some reduction in dentin elasticity, its overall effect was significantly less aggressive compared to NaOCl. Treatment with 17% EDTA resulted in severe surface degradation, precluding further mechanical assessment.

The findings support the partial rejection of both null hypotheses and highlight the necessity of carefully selecting irrigants based on their effects on dentin integrity. Polyhexanide-based solutions may represent a safer alternative for maintaining mechanical stability in clinical practice. However, additional studies involving long-term observations and larger sample sizes are needed to confirm these results. Future research should also explore the potential cumulative effects of repeated irrigation cycles on dentin structure.

## Figures and Tables

**Table 1:** Aggregated data on the effects of a polyhexanide-based antiseptic composition on dentin microhardness and associated mechanical properties.

Parameter	Treatment	Status	Min	Q1	Median	Q3	Max
**Vickers microhardness (HV)**	NaCl 0.9%	Before treatment	56.34	60.26	63.28	65.5	67.96
**Vickers microhardness (HV)**	NaCl 0.9%	After treatment	61.29	63.77	65.47	66.5	67.7
**Vickers microhardness (HV)**	NaOCl 3%	Before treatment	52.44	55.94	65.98	79.6	90.19
**Vickers microhardness (HV)**	NaOCl 3%	After treatment	52.91	54.19	60.34	68.22	70.81
**Vickers microhardness (HV)**	Chlorhexidine 2%	Before treatment	60.77	63.33	68.67	72.23	74.79
**Vickers microhardness (HV)**	Chlorhexidine 2%	After treatment	62.09	66.46	68.43	69.21	70.21
**Vickers microhardness (HV)**	EDTA 17%	Before treatment	70.04	73.97	74.55	75.33	76.78
**Vickers microhardness (HV)**	Lavasept 0.1%	After treatment	51.62	60.04	69.93	79.03	83.49
**Vickers microhardness (HV)**	Lavasept 0.1%	Before treatment	54.8	56.03	67.09	76.06	77.28
**Vickers microhardness (HV)**	Lavasept 0.2%	After treatment	62.54	64.0	65.02	69.9	76.47
**Vickers microhardness (HV)**	Lavasept 0.2%	Before treatment	62.18	63.83	64.41	65.07	65.72
**Young’s modulus (GPa)**	NaCl 0.9%	Before treatment	16.36	16.69	17.07	17.73	18.18
**Young’s modulus (GPa)**	NaCl 0.9%	After treatment	15.17	16.8	17.17	17.79	18.11
**Young’s modulus (GPa)**	NaOCl 3%	Before treatment	13.12	15.06	16.25	18.55	20.0
**Young’s modulus (GPa)**	NaOCl 3%	After treatment	12.49	13.08	14.65	16.18	17.56
**Young’s modulus (GPa)**	Chlorhexidine 2%	Before treatment	17.65	18.04	18.55	19.62	20.11
**Young’s modulus (GPa)**	Chlorhexidine 2%	After treatment	17.07	18.13	18.22	18.86	19.43
**Young’s modulus (GPa)**	EDTA 17%	Before treatment	17.85	18.3	19.53	20.45	21.43
**Young’s modulus (GPa)**	Lavasept 0.1%	After treatment	14.5	15.65	16.87	18.04	19.04
**Young’s modulus (GPa)**	Lavasept 0.1%	Before treatment	13.6	14.22	15.93	17.04	18.88
**Young’s modulus (GPa)**	Lavasept 0.2%	After treatment	14.03	14.57	14.96	15.69	16.88
**Young’s modulus (GPa)**	Lavasept 0.2%	Before treatment	12.3	13.87	14.52	14.74	15.22
**Relative elastic deformation work (%)**	NaCl 0.9%	Before treatment	23.53	23.9	24.09	24.88	26.23
**Relative elastic deformation work (%)**	NaCl 0.9%	After treatment	22.48	23.38	24.44	25.16	26.15
**Relative elastic deformation work (%)**	NaOCl 3%	Before treatment	25.9	27.12	28.75	29.61	30.23
**Relative elastic deformation work (%)**	NaOCl 3%	After treatment	23.73	24.15	24.78	25.43	26.71
**Relative elastic deformation work (%)**	Chlorhexidine 2%	Before treatment	23.69	23.97	24.51	25.94	26.95
**Relative elastic deformation work (%)**	Chlorhexidine 2%	After treatment	22.12	24.18	24.81	25.13	26.0
**Relative elastic deformation work (%)**	EDTA 17%	Before treatment	23.43	24.12	24.76	25.21	26.55
**Relative elastic deformation work (%)**	Lavasept 0.1%	After treatment	23.35	25.29	26.11	26.93	27.76
**Relative elastic deformation work (%)**	Lavasept 0.1%	Before treatment	22.6	23.52	24.22	25.48	28.13
**Relative elastic deformation work (%)**	Lavasept 0.2%	After treatment	27.2	27.35	27.63	29.94	30.47
**Relative elastic deformation work (%)**	Lavasept 0.2%	Before treatment	25.5	25.94	26.47	27.62	28.99

**Table 2:** Results of the comparison of dentin slice mechanical properties before and after treatment with the tested chemical agents.

Parameter	Treatment	*p*-Value	*p*-Value (Bonferroni Corrected)	Pseudomedian Difference	95% CI Lower	95% CI Upper
**Vickers microhardness (HV)**	NaCl 0.9%	0.1903	0.3816	+2.07	−0.94	+5.61
**Vickers microhardness (HV)**	NaOCl 3%	0.1431	0.3816	−4.49	−20.94	+8.39
**Vickers microhardness (HV)**	Chlorhexidine 2%	0.6842	0.7895	−0.98	−5.09	+3.88
**Vickers microhardness (HV)**	EDTA 17%	No data	No data	No data	No data	No data
**Vickers microhardness (HV)**	Lavasept 0.1%	0.2475	0.3816	−3.15	−20.54	+14.24
**Vickers microhardness (HV)**	Lavasept 0.2%	0.2799	0.3816	−1.54	−6.47	+0.60
**Young’s modulus (GPa)**	NaCl 0.9%	0.9698	0.9698	−0.05	−0.97	+0.73
**Young’s modulus (GPa)**	NaOCl 3%	0.1230	0.3816	−1.89	−4.09	+0.63
**Young’s modulus (GPa)**	Chlorhexidine 2%	0.2799	0.3816	−0.36	−1.50	+0.37
**Young’s modulus (GPa)**	EDTA 17%	No data	No data	No data	No data	No data
**Young’s modulus (GPa)**	Lavasept 0.1%	0.2176	0.3816	−1.16	−2.67	+0.81
**Young’s modulus (GPa)**	Lavasept 0.2%	0.0753	0.3763	−0.87	−2.06	+0.04
**Relative elastic deformation work (%)**	NaCl 0.9%	0.7959	0.8528	−0.18	−1.32	+1.02
**Relative elastic deformation work (%)**	NaOCl 3%	<0.0001	0.0006	−3.70	−4.94	−2.01
**Relative elastic deformation work (%)**	Chlorhexidine 2%	0.6842	0.7895	−0.32	−1.64	+0.97
**Relative elastic deformation work (%)**	EDTA 17%	No data	No data	No data	No data	No data
**Relative elastic deformation work (%)**	Lavasept 0.1%	0.1903	0.3816	−1.40	−2.99	+0.52
**Relative elastic deformation work (%)**	Lavasept 0.2%	0.0355	0.2660	−1.48	−3.05	−0.27

## Data Availability

The original contributions presented in this study are included in the article. Further inquiries can be directed to the corresponding author.
